# Metabolomics-based study of the effect of dietary N-carbamoylglutamic acid addition to heifers in late pregnancy on newborn calves

**DOI:** 10.3389/fvets.2024.1335897

**Published:** 2024-02-12

**Authors:** Jiandong Wang, Youli Yu, Yanan Guo, Yansheng Guo, Xiaojun Liang

**Affiliations:** ^1^Institute of Animal Science, Ningxia Academy of Agricultural and Forestry Sciences, Yinchuan, China; ^2^College of Agriculture, Ningxia University, Yinchuan, China

**Keywords:** late-gestation, calves, metabolomics, NCG, phospholipid metabolism

## Abstract

It has been demonstrated that supplementing late-gestation cow diets with NCG (N-carbamoylglutamic acid) increases the serum protein level, boosts immunological function, and increases the birth weight of the calves. However, the underlying mechanism remains unclear. In this experiment, 30 late-gestation Angus heifers almost at same conditions were chosen for this experiment. They were randomly divided into two groups of 15 cows each. A basal diet was provided to the control group, and 30 g/(d-head) of NCG was added to the basal diet of the test group (NCG group). Blood samples were collected from the jugular vein after birth and before the end (when the calves were 90 days old) of the experiment for plasma metabolomics analysis. The metabolomics analysis identified 53 metabolites between the NCG group and control group, with 40 significantly up-regulated and 13 significantly down-regulated. Among them, 33 lipids and lipid-like molecules made up 57.89% of all the metabolites that were found. Thirty-three metabolic pathways enriched by metabolites showed p.adjust <0.05, among which glycerophospholipid and sphingolipid metabolism pathways were the most abundant. In conclusion, the addition of NCG in late-gestation cows appears to primarily affect calf growth and development through the regulation of phospholipid metabolism, which plays a role in nerve conduction, brain activity, and cell metabolism and function. This study provides valuable insights into how nutritional supplementation by late-gestation cows might improve the growth and development of newborn calves.

## 1 Introduction

Arginine (Arg) plays key roles in animal reproduction and healthy. Prezotto et al. (1) showed that mothers can cope with feed deficiencies during pregnancy by modulating nutrient partitioning and altering the growth rate and function of major fetal organs. Some studies have reported that malnutrition in late gestation ewes can even lead to fetal liver fibrosis, dysfunction and antioxidant imbalance (2). Arginine (Arg) can be catalyzed by nitric oxide synthase (NOS) and ornithine decarboxylase to produce biologically active nitric oxide (NO) and polyamines. NO promotes the development of placental vasculature and its blood and nutrient supply to the embryo/fetus, it also supports the development of the placental vasculature and its blood and food supply to the embryo/fetus (3). Polyamines can act on the rate of fetal DNA and protein synthesis and regulate cell proliferation and differentiation (4). Liu et al. (5) found that the addition of 0.83% arginine to the diet of Huanjiang pig in early gestation increased the activity of NOS in the placenta and produced more NO. In addition, injection of arginine HCl in pregnant ewes not only reduced embryonic loss (6), but also increased lamb birth weight (7). By lowering the amount of dry matter, crude protein, and crude fat in the mother placenta, arginine supplementation improves the placenta’s material exchange function and facilitates the embryo’s or fetus’s effective transfer of nutrients, blood, and oxygen (3).

N-carbamylglutamate (NCG), as a precursor of Arg, promotes endogenous synthesis of Arg in animals, and even low doses of NCG are highly sensitive in increasing endogenous synthesis of Arg (8). Consequently, NCG plays a similar role in boosting animal immune system and reproductive efficiency (9, 10) and does not antagonize other amino acids (11). A previous study found that the addition of 30 g/(d-head) of NCG to the diet of late gestation heifers was able to significantly increase the calves newborn weight, increase the serum protein content of newborn calves, and improve the immune functions (12).

Metabolome refers to all small molecule compounds involved in biological processes such as metabolism and normal growth and development of an organism. Metabonomics is the systematic description of metabolite profile changes in organisms, comprehensively measuring amino acids, fatty acids and other metabolites by mass spectrometry and other assays, screening significant differences in metabolites by bioinformatics methods, and further exploring the phenotypic traits affected by these metabolites (13). Because metabolomics is the closest to phenotypic traits, it is widely used in life science research. Currently, the commonly used metabolomics studies can be divided into non-targeted metabolomics and targeted metabolomics. Non-targeted metabolomics mainly screens and annotates all the metabolites in the organism. Metabolomic measurements are usually performed on gas chromatography–mass spectrometry (GC–MS), liquid chromatography-mass spectrometry (LC–MS) and ultra performance liquid chromatography-mass spectrometry (UPLCMS) platforms (14). Metabolome has used to identified metabolites associated with color and oxidative stability of beef (15) and immune in dairy cattle (16).

In order to further study the mechanism of NCG functioning on newborn calves through pregnant cow, this experiment collected plasma from calves delivered by pregnant cows under the two conditions of adding NCG or not and to check differences in metabolites using metabolomics methods. All in all, this study provides a reference for the use of NCG as a feed additive in animal husbandry.

## 2 Materials and methods

### 2.1 Animals and study design

The experiment was conducted in Angus calves at Benwang Beef Cattle Breeding farm (Yinchuan, China). Thirty late-gestation Angus cows, nearing the anticipated date of parturition, weighing 625 ± 25 kg, and with parity 3 ~ 4 were chosen for this experiment. Previous showed that the body weight of calves was significantly higher than control with addition of 30 g/(d-head) of NCG to cows’ diet (12). Therefore, the cows in the control group were fed a basal diet, while the test group (NCG group) was designed to add 30 g/(d-head) of NCG (purchased from Linzhou Asia-Pacific Xingmu Science and Technology Co., Ltd., NCG content ≥90.0%) to the basal diet. The experiment lasts for 120 days, with a 10-day pre-feeding. Beginning four weeks before delivery, the experiment continues until the calves were 90 days old.

### 2.2 Experimental diets and feeding management

On the farm, the chosen cows were kept in separate pens with exercise yards. Following the aforementioned experimental design, each group received the diet as total mixed ration (TMR) twice a day with unrestricted access to food and water. Prior to feeding, NCG was pre-mixed with concentrates. The diet plans were developed in accordance with NRC (2016) (17) standards for beef cattle feeding, and the proportions of the basal diet composition and nutrient levels are shown in Table 1.

**Table 1:** Proportionate composition and nutrient levels of basal diets for cattle (dry matter basis, %).

Ingredient composition	Content %	Nutritional level	Value
Corn	12.25	Combined net energy (MJ/kg)	4.85
Soybean meal	3.19	Crude protein %	11.29
Cottonseed meal	2.25	Neutral detergent fiber NDF	42.25
Canola meal	3.87	Acid detergent fiber ADF	29.85
Bran	5.07	Calcium %	0.72
Sodium bicarbonate	0.30	Phosphorus %	0.35
Salt	0.27		
Premix	1.70		
Whole maize silage	51.72		
Wheat straw	19.38		
Total	100.00		

Each kg of premixed ration provides: VA: 80 KIU, VD 8: KIU, VE: 300 IU, Fe: 500 mg, Mn: 750 mg, Zn: 750 mg, Cu: 200 mg.

Following birth, care was taken to make sure the calves have enough and on time colostrum. From 5 to 90 days of age, they were also fed *ad libitum* full-price compound pellet feed and alfalfa hay. The guaranteed values of the composition of compound feed for calves are shown in Table 2.

**Table 2:** Guaranteed values for compositional analysis of calf compound feed products.

Item	Crude protein	Crude fiber	Crude ash	Calcium	Total phosphorus	Sodium chloride	Moisture	Lysine
Content (%)	≥20	≤8.0	≤10.0	0.5–1.5	≥0.4	≥0.3–1.5	≤13.0	≥1.00

### 2.3 Sample collection

In experiment, blood was collected from the jugular vein twice after calf birth and before the end (calves aged 90 days) of the experiment. The blood was centrifuged at 3,000 r/min for 15 min in a procoagulant tube soon, and then 1.5 mL of the upper serum layer was extracted by pipette and packed into freezing tubes, and stored in a freezer at −80°C. Six of each of the control group and the test group were selected for the metabolomics analysis of the blood plasma.

### 2.4 Metabolomics analysis

#### 2.4.1 Sample preparation and determination

The 100 μL of supernatant was thoroughly mixed with 400 μL of cold methanol-acetonitrile (v/v, 1:1) by vortexing. The mixture was first sonicated in an ice bath for 1 h, then incubated at −20°C for 1 h. Finally, after centrifugation at 14,000 g for 20 min at 4°C, the supernatant was collected and dried under vacuum LC–MS analysis.

The dried samples were subjected to metabolomics using a UPLC-ESI-Q-Orbitrap-MS system (UHPLC, Shimadzu Nexera X2 LC-30 AD, Shimadsu, Japan) and Q-Exactive Plus (Thermo Scientific, San Jose, United States) for metabolomics analyses. Electrospray ionization (ESI) was used for mass spectrometry data acquisition in positive and negative modes, respectively. The power conditions were set as follows: spray voltage: 3.8 kv (positive) and 3.2 kv (negative) capillary temperature: 320°C; sheath gas (nitrogen) flow rate: 30 arb (arbitrary units); auxiliary gas flow rate: 5 arb; probe heater temperature: 350°C; and S-lens RF level: 50.

#### 2.4.2 Quality control of mass spectrometry data

Peak area extraction, retention time correction, and peak comparison were performed on raw MS data using MS-DIAL. Accurate mass (mass tolerance <10 ppm) and MS/MS data (mass tolerance <0.02 Da) were used to identify the metabolites. These data sets were then compared to publicly available databases including HMDB, massbank, and independently constructed metabolite standard libraries. Only variables with more than 50% non-zero measurements in at least one group were retained in the extracted ion signature.

#### 2.4.3 Screening metabolites

The model was verified using Orthogonal Partial Least Squares Discrimination Analysis (OPLS-DA) with a threshold of *p* < 0.05 and a Variable Importance for the Projection (VIP) of >1.0. When the model was failed, the significance of metabolite changes between the two groups of samples was identified by univariate Fold Change Analysis (FC Analysis), *t*-test, and the threshold of FC ≥ 1.5 or FC ≤ 1/1.5, and *p* < 0.05. This allowed for the identification of the significance of metabolite changes between the NCG group and the control group.

#### 2.4.4 KEGG enrichment analysis

KEGG pathway analysis was performed on the identified metabolites using the KEGG database.^1^ The overall pathway importance was calculated based on the sum of the importance index (ImpactODC) of each matched metabolite divided by the sum of the importance measures of all metabolites in each pathway. The closer the ImpactODC is to 1, the more pronounced the identified metabolite effect is. We therefore define the significant KEGG pathway as *p* < 0.05 or ImpactODC >0.

#### 2.4.5 Statistical analysis

Since we were divided into different experimental and treatment groups, one-way ANOVA was more suitable for analyzing our data and detecting the differences between the different groups. Therefore, the data were statistically analyzed by one-way ANOVA using R (version:4.0.3), with *p* < 0.05 indicating a statistically significant difference.

## 3 Results

### 3.1 Partial least squares discriminant analysis

**Figure 1:**
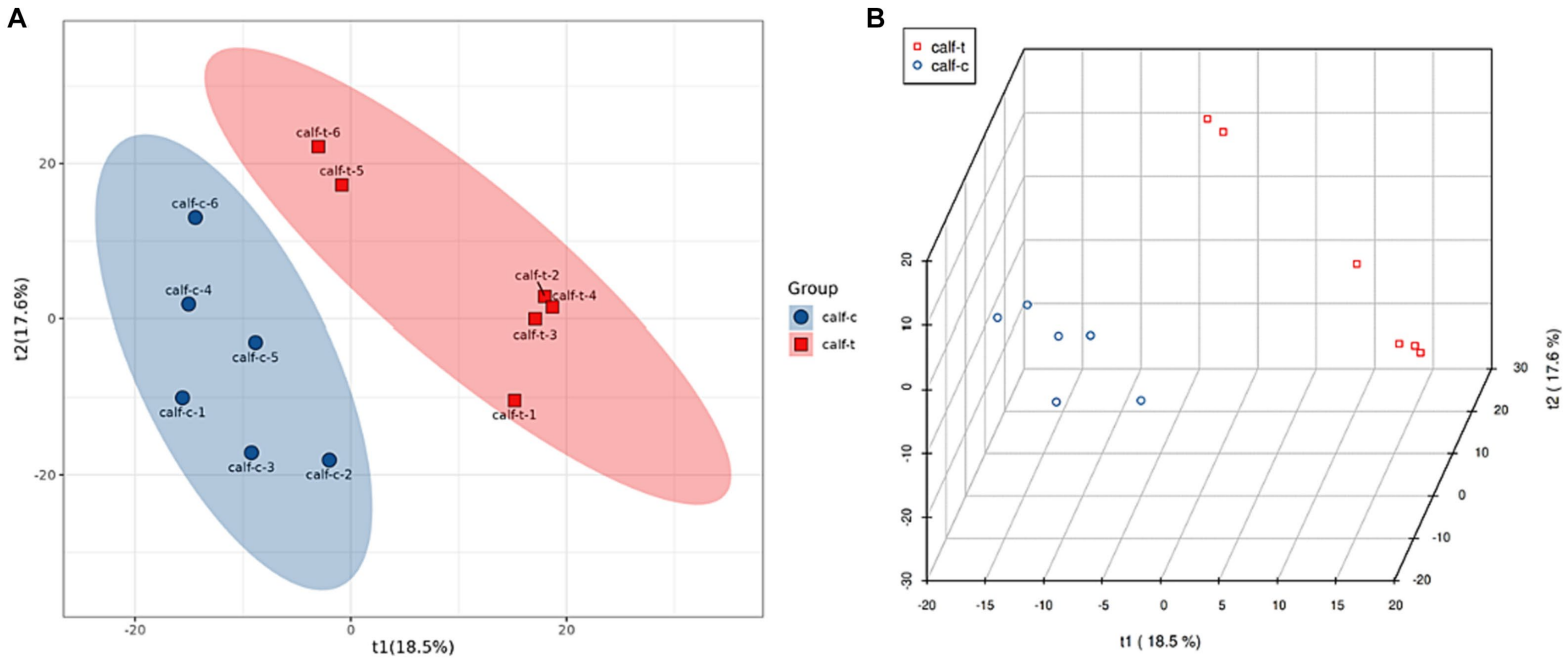
Plot of PLS-DA scores **(A)** and PLS-DA 3d scores **(B)** for the test group and control group.

The Partial Least Squares Discrimination Analysis (PLS-DA) model was established for the NCG group compared with the control group, and the evaluation parameters of the model can be seen from Table 3. R2Y > 0.99 and R2X > 0.59 indicate that the model is stable and reliable, and Q2 > 0.6 indicates that the model’s predictive ability is good. As can be seen from Figure 1, there were intergroup differences between the NCG group and the control group in the first principal component of principal components analysis.

**Table 3:** Parameters of PLS-DA model evaluation between NCG group vs. control group.

R2X(*cum*)	R2Y(*cum*)	Q2(*cum*)	RMSEE	pre	ort	pR2Y	pQ2
0.596	0.997	0.678	0.0348	4	0	0.87	0.39

R2X and R2Y denote the explanatory rate of the model on the X and Y matrices; Q2: denotes the predictive ability of the model; the closer R2 and Q2 are to 1, the more stable and reliable the model is. Q2 > 0.5 indicates a good predictive ability of the model, Q2 less than 0.5 indicates a poor predictive ability of the model; RMSEE: Root Mean Square Error of Estimation; pre denotes the prediction group score used for modeling, ort denotes the orthogonal group score used for modeling; pR2Y: *p*-value for R2Y, pQ2: *p-*value for Q2.

### 3.2 Orthogonal partial least squares discriminant analysis

The results of OPLS-DA from Supplementary Table S1 show that R2X and Q2 are less than 0.5, while R2Y is nearly equal to 1. The model is stable and reliable on the Y-axis, but relatively unstable on the X-axis, and the model predictive ability is poor, the modeling fails. Therefore, FC ≥ 1.5 or FC ≤1/1.5 with *p* < 0.05 were used to identify differential metabolites.

### 3.3 Screening differential metabolites

Using both positive and negative ion modes, a total of 1,262 metabolites were identified in the plasma of the NCG group vs. control group. A total of 53 metabolites were screened using FC ≥ 1.5 or FC ≤ 1/1.5 with *p* value <0.05, and the NCG group was significantly up-regulated by 40 metabolites and significantly down-regulated by 13 metabolites compared to the control group. Nine differential metabolites were identified, with five showing significant up-regulation and four showing significant down-regulation in the NCG group compared to the control group, provided the *p-*value was less than 0.01.

### 3.4 Expression analysis of the identified metabolites

**Figure 2:**
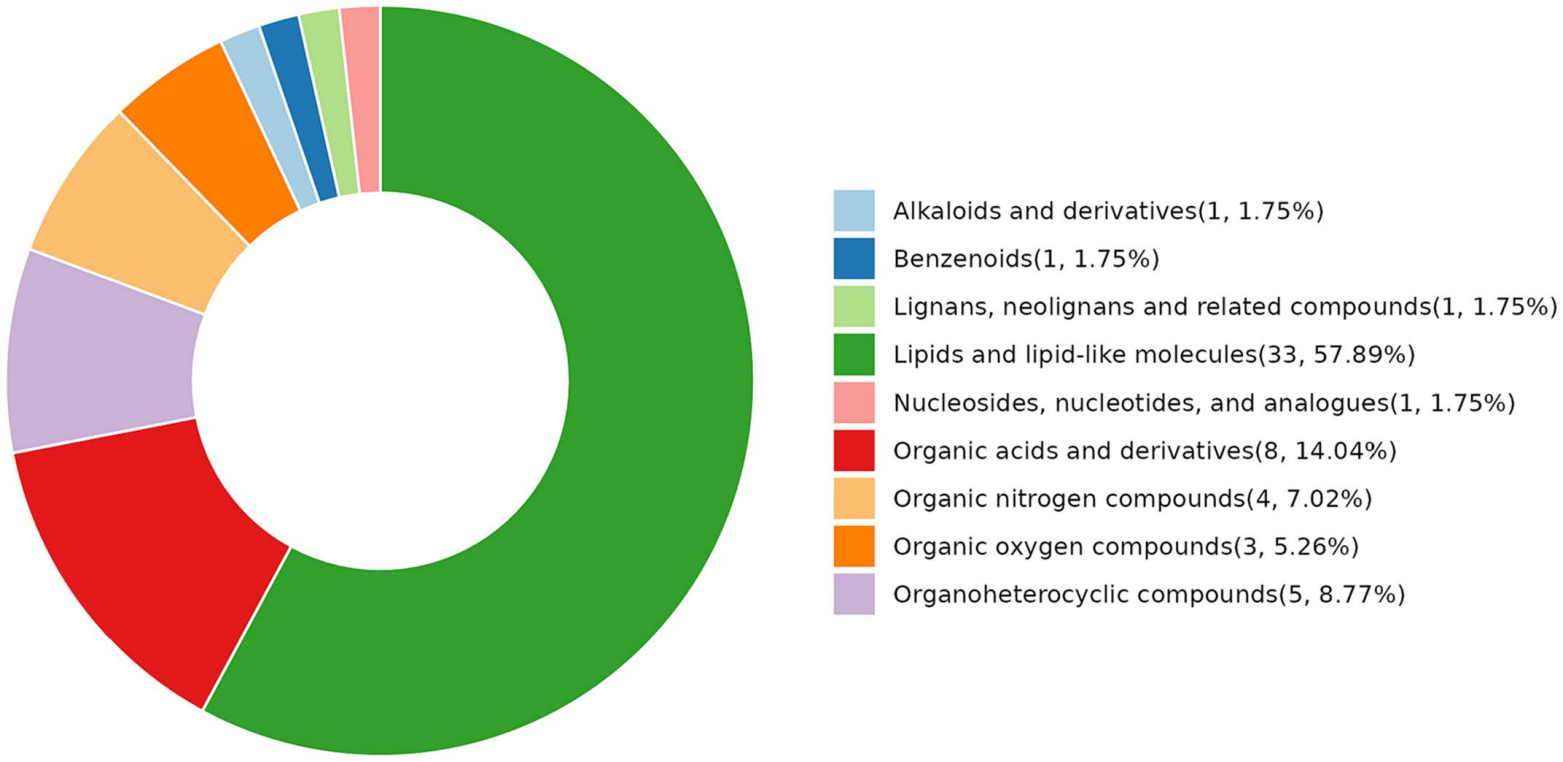
Classification ring diagram of differential metabolites between the NCG group vs. control group (HMDB Super Class).

Under the condition of *p* < 0.05, we identified 53 metabolites (Figure 2), including one metabolite belonging to alkaloids and derivatives, one belonging to benzenoids, one belonging to lignans, one belonging to neolignans and related compounds, 33 belonging to lipids and lipid-like molecules, one belonging to nucleosides, nucleotides, and analogs, eight belonging to organic acids and derivatives, four belonging to organic nitrogen compounds, three belonging to organic oxygen compounds, five belonging to organic heterocyclic compounds. The largest percentage of detected metabolites (57.89%) were lipids and lipid-like compounds, which were followed in abundance by organic acids and their derivatives. Other types of differentiated metabolites were comparatively less abundant.

### 3.5 Functional analysis of identified metabolites

**Figure 3:**
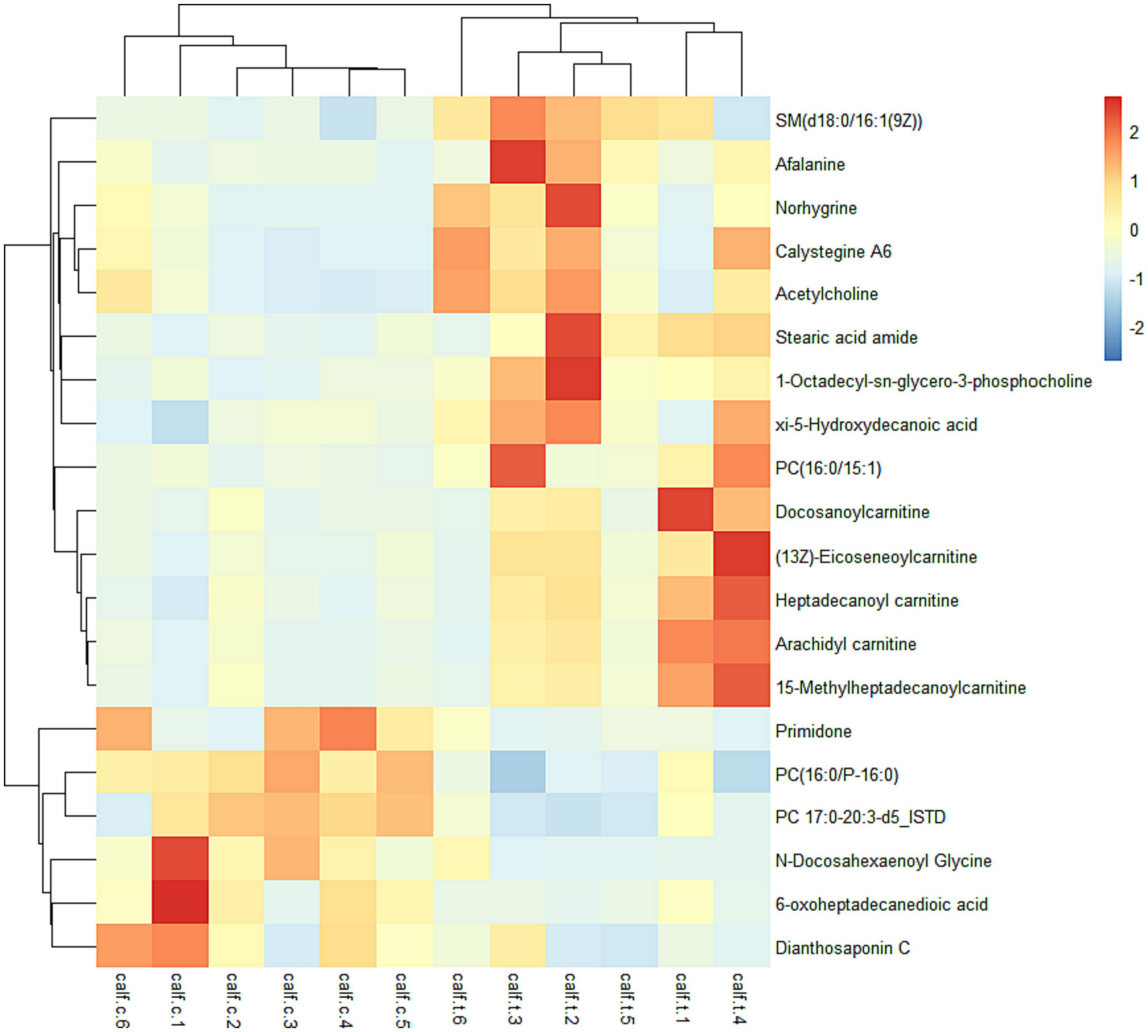
The identified top 25 metabolites in NCG group vs. control group.

We conducted association analysis using metabolites with weight reported in our previous work (12), and results suggested that 16 metabolites were associated with weight of days 0 and 90 (Supplementary Figure S1). The result highlights the important roles of metabolites in calves’ growth. The screened metabolites (Figure 3) were enriched in 33 metabolic pathways, of which 21 had *p* < 0.05, including glycerophospholipid metabolism, regulation of the actin cytoskeleton, cholinergic synapses, sphingomyelin signaling pathway, neuroactive ligand-receptor interaction, apoptosis, bile secretion, and phospholipase D signaling pathway. As shown in Table 4, glycerophospholipid metabolism and sphingolipid metabolism pathways have *p.adjust* < 0.05. The results of pathway enrichment showed that the effect of identified metabolites in glycerophospholipid metabolism and sphingolipid metabolism pathways was most significant in the NCG group vs. the control group.

**Table 4:** The identified metabolite-enriched metabolic pathways in NCG group vs. control group.

Pathways	*P*-value	*P* adjust value	ImpactODC
Glycerophospholipid metabolism	0.000	0.000	0.113
Sphingolipid metabolism	0.001	0.006	0.083
Ether lipid metabolism	0.050	0.078	0.000
Glycerolipid metabolism	0.075	0.097	0.042
Glycine, serine and threonine metabolism	0.093	0.107	0.019
Fatty acid degradation	0.097	0.107	0.012
Fatty acid metabolism	0.222	0.237	0.008
Metabolic pathways	0.969	0.969	0.004

**Figure 4:**
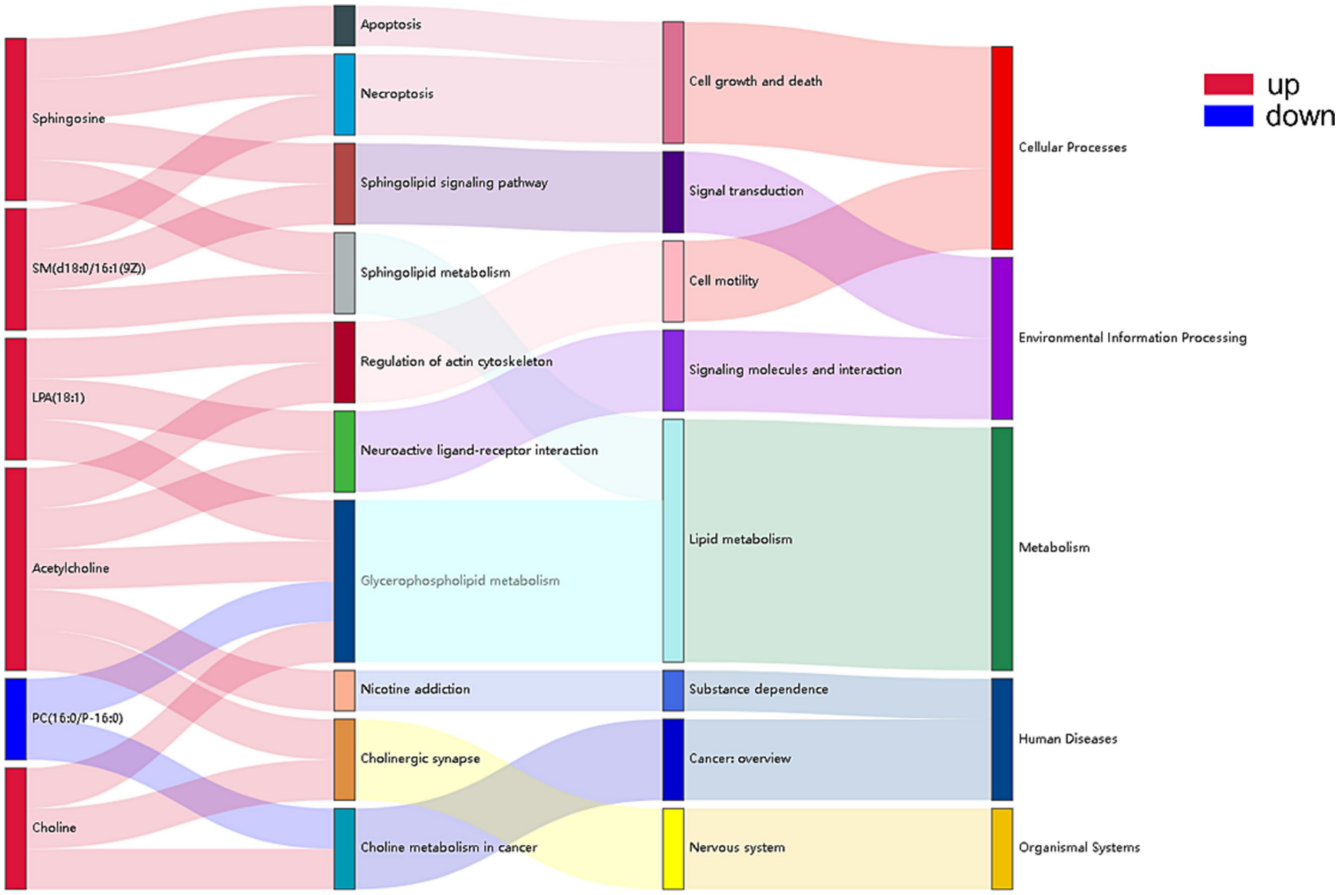
Sankey diagram of metabolic pathways enriched for identified metabolites between NCG group and control group. Pathways enriched for significance top15 in relation to differential metabolites, from left to right are differential metabolites (red indicates up-regulation, blue indicates down-regulation), significantly enriched (*p* < 0.05) metabolic pathways, KEGG pathway categories, and the top KEGG pathway categories.

A mulberry diagram was created using the top 15 metabolic pathways where there were significant differences between the NCG and control groups (Figure 4). It suggested that the down-regulation of phosphatidylcholine (16:0/P-16:0) is involved in glycerophospholipid metabolism and cholinergic metabolism in cancer. In addition, sphingomyelin, sphingomyelin [d18:0/16:1(9Z)], lysophosphatidic acid (18:1), acetylcholine, and choline has an impact on glycerophospholipid metabolism, cholinergic synapses, the sphingomyelin signaling pathway, cholinergic synapses, sphingolipid metabolism, neutrally active ligand-receptor interactions were all up-regulated.

## 4 Discussion

Phospholipids are composed of 3-phosphoglycerol, and there are two main types of phospholipids, glycerophospholipids (Glycerol phospholipid, GPC) and sphingomyelin (sphingomyelin, SM) (18). Phospholipids are an essential part of cell membranes and can be used as a significant source of accessible carbon in the body for processes like Mycoplasma parasitism. They also play important roles in improving memory, boosting fat metabolism, and promoting nerve conduction and brain function (19). In this study, 33 metabolites of lipid and lipid-like molecules were found in the NCG group vs. the control group, accounting for 57.89% of the totally different metabolites. The metabolic pathways with the highest significance index and statistically significant differences were glycerophospholipid metabolism and sphingolipid metabolism. This suggests that controlling the metabolism of phospholipids is the primary mechanism by which administering NCG to cows during the late gestational stage affects the growth and development of the calf. The phospholipids, as an important constituent of cell membranes, has a fundamental role in cell metabolism and function, and even more so in the activation of signaling pathways.

Lysophosphatidic acid (LPA) is a single-chain fatty acylphospholipid derivative produced by hydrolysis or enzymatic hydrolysis of the ester bond at the 1- or 2-position of phospholipids. It can be produced either during the biosynthesis of cytosolic phospholipids or by activated platelets, leukocytes, endothelial cells, neuronal cells and tumor cells, etc. (20). In a preliminary research of T cells, Van Kempen et al. (21) verified the driving action of LPA on lymphocytes, as well as its capacity to promote the proliferation of Jurkat T cells and elevate the secretion of interleukin 2 (IL-2). Furthermore, LPA inhibited apoptosis caused by anti-Fas, CD2, CD3, and CD28 antibodies in CD4+ 8+ 3 low T lymphoblastoid cells of the Tsup-1 lineage when it was present as a survival factor in blood-borne mouse macrophages (22, 23). The down-regulation of the apoptosis-promoting protein, Bax, induced by LPA, may be responsible for the anti-apoptotic effect. In addition, LPA increased the migration of macrophages that resembled histiocytes and shown increased cell proliferation in *in vivo* (24), and is an important growth factor in the healing process. SM, PC, and LPC all contain choline, and acetylcholine is synthesized from choline and acetyl-coenzyme A catalyzed by choline acetyltransferase. T-cell-derived acetylcholine (ACh) has often been extensively studied as a neurotransmitter but has also been found to play an important role in the regulation of immunity. When mice were infected with the lymphocytic choroidal meningitis virus, Cox et al. (25) found that choline acetyltransferase (ChAT) was significantly elevated in an IL-21-dependent way in CD4+ and CD8+ T cells. This, in turn, stimulated the formation of ACh. Nonetheless, when Chat was missing from the T-cell compartment, both the synthesis of ACh and the antiviral T cell’s migration to the infected tissues affected, indicating that ACh and immunomodulation are positively correlated. In this experiment, lysophosphatidic acid, choline, and acetylcholine in the glycerophospholipid metabolic pathway were significantly up-regulated between NCG group vs. control group, and sn-Glycero-3-phosphocholine was significantly down-regulated. Oxidation products of 2-arachidonic-1-palmitoyl-sn-glycero-3-phosphocholine are involved in chronic inflammation and vascular diseases (26). The significant difference of metabolites in the glycerophospholipid metabolic pathway indicates that calves in the NCG group had higher immunocompetence and a reduced incidence of illness. This result supports the significant elevation of serum IgA, IgG, and IgM levels in newborn calves found in the previous period when NCG was added to the diets of late gestation heifers at 30 g/(d-head) (12). It was demonstrated that calves in the NCG group secreted more metabolites that have the capacity to modulate the immune system, such as choline, acetylcholine, and lysophosphatidic acid, through synthesis, and these metabolites have a role in boosting the organism’s immunity and increasing the survival rate. Similarly, sphingolipids are important components of cell membranes, mainly including ceramides, sphingomyelins, and 1-phospho-sphingosine. Sphingolipids function as intracellular messengers in a variety of biological processes, including cell development, death, proliferation, and invasion. They can also control protein activity and activate signaling pathways (27). The changes in levels of different types of sphingolipids can lead to important changes in overall cellular function. Among them, sphingolipids exert pleiotropic effects on protein kinases and other targets, and play important roles in the regulation of the actin cytoskeleton, cytophagy, cell cycle and apoptosis (28). In addition, LPA, as a lipid small molecule with growth factor-like properties (29), can influence the function of target cells through a variety of signaling, promoting cell proliferation or apoptosis, platelet aggregation, etc. (30). Moreover, in the early postnatal heart, LPA3-mediated LPA signaling is essential for cardiomyocyte proliferation (31). In the absence of significant difference between upstream and downstream metabolites, our analysis revealed that sphingomyelin and sphingosine in the sphingolipid metabolic pathway were significantly up-regulated between NCG group vs. control group. The results were consistent with the significant elevation of birth weight, body weight, weight, and average daily gain of calves at 90 days of age as well as the serum TP, ALB, and NO content in the previous study (12). Consequently, the addition of 30 g/(d-head) of NCG to the diets of the late gestation cows can have a positive effect on the overall cellular and functions. The body is therefore more robust than the control group due to more active cellular growth, better regulation of protein activity, and activation of signaling pathways.

## 5 Conclusion

The metabolomics analysis in this study demonstrated that the effect of NCG supplementation to cows in late gestation on calf growth and development was mainly based on the regulation of phospholipid metabolism. The metabolites of lysophosphatidic acid, choline, and acetylcholine were upregulated and enriched in glycerophospholipid metabolism pathway. It has the ability to regulate immunological function, contributing to the body’s increased immunity and increased viability rate. This study provides a guide how to improve calf survival rate from nutrition level.

## Data availability statement

The original contributions presented in the study are publicly available. This data can be found at: www.ebi.ac.uk/metabolights/; MTBLS8983.

## Ethics statement

The animal study was approved by the Ningxia Society of Science and Technology Ethics (Ningxia, China). The study was conducted in accordance with the local legislation and institutional requirements. Written informed consent was obtained from the owners of Benwang Beef Cattle Breeding farm to allow animals to participate in this study.

## Author contributions

JW: Conceptualization, Formal analysis, Methodology, Project administration, Writing – original draft, Writing – review & editing. YY: Conceptualization, Formal analysis, Writing – review & editing. YG: Conceptualization, Methodology, Writing – review & editing. YSG: Formal analysis, Methodology, Writing – review & editing. XL: Writing – review & editing.

## Footnotes

^1^ http://www.kegg.jp

## References

[1] Prezotto LD Camacho LE Lemley CO Keomanivong FE Caton JS Vonnahme KA. Nutrient restriction and realimentation in beef cows during early and mid-gestation and maternal and fetal hepatic and small intestinal in vitro oxygen consumption. Animal. (2016) 10:829–37. doi: 10.1017/S1751731115002645, PMID: 27087042

[2] Liu Y Li H Sha Q Hai R Wang Y Song Y. Effects of maternal undernutrition on the growth, development and antioxidant status of ovine placentome subtypes during late pregnancy. Theriogenology. (2018) 110:96–102. doi: 10.1016/j.theriogenology.2018.01.002, PMID: 29407903

[3] Bazer FW Davis TA Jaeger LA Johnson GA Kim SW Knabe DA. Important roles for the arginine family of amino acids in swine nutrition and production. Livest Sci. (2007) 112:8–22. doi: 10.1016/j.livsci.2007.07.003

[4] Igarashi K Kashiwagi K. Polyamines: mysterious modulators of cellular functions. Biochem Biophys Res Commun. (2000) 271:559–64. doi: 10.1006/bbrc.2000.2601, PMID: 10814501

[5] Liu JF Chen W Kong XF Yang HS Li FN Geng MM. Effects of dietary supplemented with arginine on growth and development of fetus in pregnant Huanjiang mini-pigs. Sci Agric Sin. (2011) 44:1040–5. doi: 10.3864/j.issn.0578-1752.2011.05.022

[6] Uyanga VA Xin Q Sun M Zhao J Wang X Jiao H. Research note: effects of dietary l-arginine on the production performance and gene expression of reproductive hormones in laying hens fed low crude protein diets. Poult Sci. (2022) 101:101816. doi: 10.1016/j.psj.2022.101816, PMID: 35339936 PMC8957049

[7] Lassala A Bazer FW Cudd TA Datta S Keisler DH Satterfield MC. Parenteral administration of L-arginine enhances fetal survival and growth in sheep carrying multiple fetuses. J Nutr. (2011) 141:849–55. doi: 10.3945/jn.111.138172, PMID: 21430253 PMC3078019

[8] Ma N Li Y Ren L Hu L Xu R Shen Y. Effects of dietary N-carbamylglutamate supplementation on milk production performance, nutrient digestibility and blood metabolomics of lactating Holstein cows under heat stress. Anim Feed Sci Tech. (2021) 273:114797. doi: 10.1016/j.anifeedsci.2020.114797

[9] Hu N Ma WF Mao P Wu QJ. Research progress of n-carbamoyl glutamate in alleviating intestinal oxidative stress in pigs. Chin J Anim Nutr. (2023) 35:6. doi: 10.12418/CJAN2023.195

[10] Zhao HX Qiao GX Li PJ Chen XY Chen B Huang W. Effects of dietary L-arginine or N-Carbamylglutamate on growth performance, intestinal function, serum biochemical indexes and anti-ammonia-nitrogen stress ability of yellow catfish (*Pelteobagrus fulvidraco*). Chin J Anim Nutr. (2021) 33:6330–9. doi: 10.3969/j.issn.1006-267x.2021.11.033

[11] Peng Y Yang HS Wu X Li L Yin YL. Application of N-Carbamoylglutamate in swine nutrition. Chin J Anim Nutr. (2013) 25:1131–6. doi: 10.3969/j.issn.1006-267x.2013.06.001

[12] Chen ZL Zeng YX Wang BL Chen CJ Zhu XZ Yang XY. Effect of NCG supplementation in diet of Angus cows on growth performance, serum biochemical indexes and immune indexes of calves. Feed Res. (2023) 15:1–4. doi: 10.13557/j.cnki.issn1002-2813.2023.15.001

[13] Aderemi AV Ayeleso AO Oyedapo OO Mukwevho E. Metabolomics: a scoping review of its role as a tool for disease biomarker discovery in selected non-communicable diseases. Meta. (2021) 11:418. doi: 10.3390/metabo11070418, PMID: 34201929 PMC8305588

[14] Johnson CH Ivanisevic J Siuzdak G. Metabolomics: beyond biomarkers and towards mechanisms. Nat Rev Mol Cell Biol. (2016) 17:451–9. doi: 10.1038/nrm.2016.25, PMID: 26979502 PMC5729912

[15] Ma D Kim YHB Cooper B Oh JH Chun H Choe JH. Metabolomics profiling to determine the effect of postmortem aging on color and lipid oxidative stabilities of different bovine muscles. J Agric Food Chem. (2017) 65:6708–16. doi: 10.1021/acs.jafc.7b02175, PMID: 28700223

[16] Joo SS Lee SJ Park DS Kim DH Gu BH Park YJ. Changes in blood metabolites and immune cells in Holstein and Jersey dairy cows by heat stress. Animals. (2021) 11:974. doi: 10.3390/ani11040974, PMID: 33807443 PMC8065422

[17] National Academies of Sciences, Engineering, and Medicine. Nutrient requirements of beef cattle. 8th Revised ed. Washington, DC: The National Academies Press (2016).

[18] Rhee SG Choi KD. Regulation of inositol phospholipid-specific phospholipase C isozymes. J Biol Chem. (1992) 267:12393–6. doi: 10.1016/0092-8674(92)90644-R, PMID: 1319994

[19] Großhennig S Schmidl SR Schmeisky G Busse J Stülke J. Implication of glycerol and phospholipid transporters in mycoplasma pneumoniae growth and virulence. Infect Immun. (2013) 81:896–904. doi: 10.1128/IAI.01212, PMID: 23297388 PMC3584876

[20] Yue H Wang Y. Effects of lysophosphatidic acid on nervous system cells and its relationship with ischaemic stroke. Chin J Stroke. (2008) 3:828–33.

[21] Van Kempen MJ Fromaget C Gros D Moorman AF Lamers WH. Spatial distribution of connexin43, the major cardiac gap junction protein, in the developing and adult rat heart. Circ Res. (1991) 68:1638–51. doi: 10.1161/01.RES.68.6.1638 1645233

[22] Koh JS Lieberthal W Heydrick S Levine JS. Lysophosphatidic acid is a major serum noncytokine survival factor for murine macrophages which acts via the phosphatidylinositol 3-kinase signaling pathway. J Clin Invest. (1998) 102:716–27. doi: 10.1172/JCI1002, PMID: 9710440 PMC508934

[23] Dolber PC Beyer EC Junker JL Spach MS. Distribution of gap junctions in dog and rat ventricle studied with a double-label technique. J Mol Cell Cardiol. (1992) 24:1443–57. doi: 10.1016/0022-2828(92)91085-J, PMID: 1338112

[24] Peters NS. New insights into myocardial arrhythmogenesis: distribution of gap-junctional coupling in normal, ischaemic and hypertrophied human hearts. Clin Sci. (1996) 90:447–52. doi: 10.1042/cs0900447, PMID: 8697713

[25] Cox MA Duncan GS Lin GHY Steinberg BE Yu LX Brenner D. Choline acetyltransferase-expressing T cells are required to control chronic viral infection. Science. (2019) 363:639–44. doi: 10.1126/science.aau9072 30733420 PMC7181845

[26] Tang WJ. Enzymatic synthesis of monoacylglycerols and symmetrical triacylglycerols rich inarachidonic acid at the sn-2 position [Master's thesis]. Wuxi: Jiangnan University (2008).

[27] Green CD Maceyka M Cowart LA Spiegel S. Sphingolipids in metabolic disease: the good, the bad, and the unknown. Cell Metab. (2021) 33:1293–306. doi: 10.1016/j.cmet.2021.06.006, PMID: 34233172 PMC8269961

[28] Hannun YA Obeid LM. Principles of bioactive lipid signalling: lessons from sphingolipids. Nat Rev Mol Cell Biol. (2008) 9:139–50. doi: 10.1038/nrm2329, PMID: 18216770

[29] Ye X Chun J. Lysophosphatidic acid (LPA) signaling in vertebrate reproduction. Trends Endocrinol Metab. (2010) 21:17–24. doi: 10.1016/j.tem.2009.08.003, PMID: 19836970 PMC2818173

[30] Zhou Z Niu J Zhang Z. The role of lysophosphatidic acid receptors in phenotypic modulation of vascular smooth muscle cells. Mol Biol Rep. (2010) 37:2675–86. doi: 10.1007/s11033-009-9798-6, PMID: 19757175

[31] Wang F Liu S Pei J Cai L Liu N Liang T. LPA3-mediated lysophosphatidic acid signaling promotes postnatal heart regeneration in mice. Theranostics. (2020) 10:10892–907. doi: 10.7150/thno.47913, PMID: 33042260 PMC7532668

