# Supplementary material for: Metabolomics-based study of the effect of dietary N-carbamoylglutamic acid addition to heifers in late pregnancy on newborn calves
Source: Front Vet Sci. 2024 Feb 12;11:1335897. doi: 10.3389/fvets.2024.1335897 (PMC10894953; doi:10.3389/fvets.2024.1335897)
Supplement: Supplementary file 1 [file Data_Sheet_1.docx]

Supplementary Material

# Supplementary Table 1. Evaluation parameters of OPLS-DA model in the test group compared to the control group.

| R2X(cum) | R2Y(cum) | Q2(cum) | RMSEE | pre | ort | pR2Y | pQ2 |
| --- | --- | --- | --- | --- | --- | --- | --- |
| 0.476 | 0.986 | 0.156 | 0.0713 | 1 | 2 | 0.525 | 0.52 |

Note: R2X and R2Y denote the explanatory rate of the model on the X and Y matrices; Q2: denotes the predictive ability of the model; the closer R2 and Q2 are to 1, the more stable and reliable the model is. Q2 > 0.5 indicates a good predictive ability of the model, Q2 less than 0.5 indicates a poor predictive ability of the model; RMSEE: Root Mean Square Error of Estimation; pre denotes the prediction group score used for modelling, ort denotes the orthogonal group score used for modelling; pR2Y: p-value for R2Y, pQ2: p-value for Q2.

# Supplementary Figure S1. The association of metabolites with weight of day 0 and day 90.


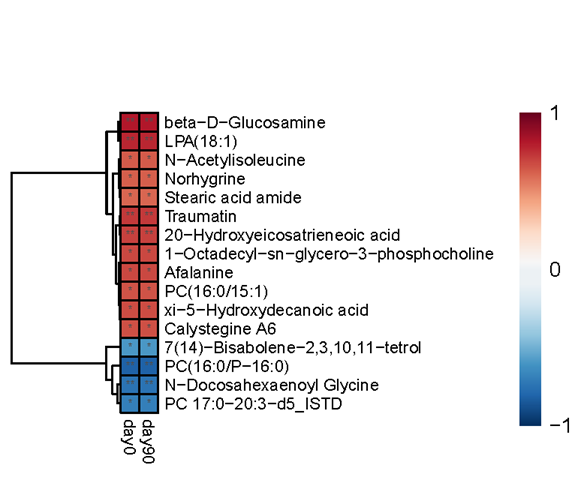


Notes: *** means *p* < 0.001; ** means *p* < 0.01; * means *p* < 0.05
